# Evaluation of Lacrimal Punctal Changes by Anterior Segment Optical Coherence Tomography after Punctal Dilation Versus Punctal Plug Insertion in Cases of Inflammatory Punctual Stenosis

**DOI:** 10.1155/2022/7666323

**Published:** 2022-10-20

**Authors:** Islam Awny, Elshimaa A Mateen Mossa, Tasneem Mohammed Bakheet, Hany Mahmoud, Amr Mounir

**Affiliations:** ^1^Sohag Faculty of Medicine, Ophthalmology Department, Sohag University, Sohag, Egypt; ^2^Sohag Faculty of Medicine, Public Health and Community Medicine Department, Sohag University, Sohag, Egypt

## Abstract

**Purpose:**

To compare simple punctal dilation versus punctal dilation augmented by insertion of Punctal Plug Insertion (PPP) with assessing the effect on resolving epiphora and punctum size improvement by an objective method, high-resolution AS-OCT imaging comparing punctal parameters of the patients before and after both techniques. *Patients and Methods*. It is a prospective, interventional, double-blinded controlled randomized study, which was conducted on two groups of patients, the first epiphora group (EG1 25 eyes), who had acquired punctal stenosis and epiphora and managed by simple punctal dilatation. The second epiphora group (EG2 20 eyes) who managed by punctal dilatation augmented with an application of perforated punctal plugs for two months. Spectral Domain Anterior Segment-OCT Image Acquisition. AS-OCT for lower puncti was performed using RTVue (Optovue Inc., Fermont, CA). OCT images of the width and length of the lower puncti of the participants were captured by the same operator on the same machine. Measuring of punctal size (diameter and length) was performed before treatment, one week, one month, and three months later objectively by using AS-OCT.

**Results:**

The study included 45 eyes of 50 subjects; the total coverage of epiphora patients fulfilled inclusion criteria and presented to the ophthalmology clinics of Sohag University Hospitals in the period between June 2021 and December 2021. Both groups were comparable regarding mean age (39 ± 11 vs 50 ± 12 years, *P* value = 0.4) and sex (males were 36% vs 40%, female were 64% vs 60%, *P* value = 0.5) respectively with no statistically significant difference between both groups. The mean duration of epiphora was measured in both groups before treatment (EG1 = 1.656 ± 0.41 months, EG2 = 1.73 ± 0.32 months) with no statistically significant difference (*P* value = 0.436). There was marked improvement of the outer punctual diameter and length between outer and inner puncti in EG1 (EG1 391um ± 122 um, EG2 692 ± 226 um (*P* value < 0.007) and EG1 189 ± 43 um, 380 ± 169 um (*P* value < 0.0002) during the follow-up period. EG2 showed more improvement than EG1 when compared during the follow-up period in both outer punctual diameter and length between outer and inner puncti with (*P* value < 0.003 and < 0.0002) in favor of EG2. However; both groups show improvement with the statistically significant difference in both groups by using AS-OCT.

**Conclusions:**

Punctal dilation augmented by insertion of PPP was an effective method in treating cases of inflammatory punctual stenosis as found by monitoring of punctal parameters changes by AS-OCT. AS-OCT was found a useful method for the evaluation of the lacrimal punctal parameters, especially with different treatment modalities in epiphora cases.

## 1. Introduction

Epiphora or tearing is a common complaint in the ophthalmology outpatient clinics with the most commonly accused disease being the acquired punctal stenosis. Reference [1] punctal stenosis can be defined as occlusion or narrowing of the outwards palpebral opening of the lacrimal canaliculi with a patent distal lacrimal drainage system. A more accurate definition is that the punctum size is less than 0.3 mm, or the inability to cannulate the punctum with a 26G cannula without being dilated. [2].

Several methods can be used for the Grading of punctal stenosis. Kashkouli's scoring system is one of the most commonly used methods (grade 0 no punctum (agenesis), grade 1 puncta papilla is covered with a membrane (difficult to recognize), and the grade 2 is less than the normal size but recognizable, grade 3 punctum is normal, grade 4 punctum is small slit (<2 mm), and grade 5 punctum is large slit (≥2 mm)). [3].

Another subjective method is, the Munk scale for epiphora grading system (grade 0 no epiphora, grade 1 epiphora requiring dabbing less than twice a day, grade 2 epiphora requiring dabbing 2–4 times a day, grade 3 epiphora requiring dabbing 5–10 times a day, grade 4 epiphora requiring dabbing more than 10 times a day, and grade 5 is constant epiphora). [4].

There are various currently used treatment options for the acquired punctal stenosis, including simple repeated combined dilatation of the puncta and canaliculi to overcome any fibrosis or stenosis. Punctal enlargement by dilatation with insertion of adjuvants as (perforated punctal plugs (PPP), mini-monoka canalicular stents (MMC), or self-retaining bicanalicular stents (SRBC)). Punctal dilation and snip incisions, which can also be augmented by insertion of (MMC). [5].

Anterior segment optical coherence tomography (AS-OCT) is a noninvasive, safe, accurate, objective, and cross-sectional imaging method used to study the anterior segment of the eye including parameters of lacrimal puncta, cornea, conjunctiva, angle of the anterior chamber, and the lower tear meniscus height. [6, 7].

The limited number of studies in the literature that compared the different techniques for the treatment of epiphora resulted in absence of any consensus on which procedure is the best for the treatment of such cases.

This work aimed to compare simple punctal dilation versus punctal dilation augmented by insertion of PPP with assessing the effect on resolving epiphora and punctum size improvement by an objective method, high-resolution AS-OCT imaging comparing punctal parameters of the patients before and after both techniques.

## 2. Patients and Methods

The study included 45 eyes of 50 patients of epiphora patients who fulfilled the inclusion criteria and presented to the ophthalmology clinics of Sohag University Hospitals in the period between June 2021 and December 2021.

Patients included in this research were 20 years old or older who had acquired inflammatory punctal edema and were complaining of epiphora and did not respond to medical treatment of epiphora. Punctal stenosis grading of the included groups was graded 1 : 4 according to Kashkouli's classification [3], and epiphora grading was 3 : 5 according to Munk's classification [4].

Patients with a history of previous ocular or lacrimal surgical intervention, trauma, glaucoma disease, corneal abnormalities (abrasions and keratitis), congenital punctal anomalies, and congenital nasolacrimal duct obstruction were excluded.

The study design is a prospective, interventional, double-blinded controlled randomized study, which was conducted on two groups of patients, the first epiphora group (EG1) included 25 eyes, who had acquired punctal stenosis, and epiphora and managed by simple punctal dilatation. The second epiphora group (EG2) included 20 eyes who managed by punctal dilatation augmented with the application of perforated punctal plugs for two months.

In EG1, the procedures were performed under topical anesthesia using proparacaine chloride, the punctum was located using a punctal finder, and punctal dilation and probing, and irrigation were performed using a 26 G lacrimal cannula in the operating room. Patients who were diagnosed as punctal stenosis as a result to canalicular membranous obstruction or a soft stop during probing that could not be overcome were excluded from the study.

In EG2 after completing the procedure done in EG1, silicone plugs (FCI's PVP PRELOADED PERFORATED PLUGS, FCI Ophthalmics, Paris, France) were implanted using their preloaded inserter. They perform their functions by acting like conformers by maintaining the punctal opening dilated for several weeks allowing tears to be drained. These plugs are coated with a thin layer of polyvinylpyrrolidone (PVP) to increase the hydrophilic properties of silicone. The final position of the plug was checked to ensure an appropriate fit within the lid margin and explanted after 2 months. Postoperatively in both procedures, topical antibiotics and steroid eye drops were administered for 1 week.

Patients' selection was based on slit-lamp examination of the eyelids, corneal surface, bulbar and palpebral conjunctiva, tear meniscus, intraocular pressure (IOP) measurement, lower punctal examination, and measuring of punctal size (diameter and length) before treatment, one week, one month, and three months later objectively by using AS-OCT.

Spectral Domain Anterior Segment-OCT Image Acquisition. AS-OCT for lower puncti was performed using RTVue (Optovue Inc., Fermont, CA). OCT images of the width and length of the lower puncti of the participants were captured by the same operator on the same machine. The lower eyelid margin was gently everted using a cotton bud that was placed below the punctum. The punctum was everted perpendicular to the light source to allow alignment of the punctum depth concerning the axis of the scanner's infrared beam. The punctum was imaged with the scan line placed horizontally along the mucocutaneous junction. Outer punctal diameter was measured as the distance between the two highest points on the nasal and temporal punctal orifice. Punctal depth is the vertical lumen; it was measured vertically between outer and inner punctal openings which appear closed.

This study was approved by the Ethical Committee of Faculty of Medicine, Sohag University, under IBR registration number *S20-160* with clinical trial registration number *PACTR202105834743189* and was performed according to the Declaration of Helsinki. The aim of the study and intervention details were discussed with the participants and informed written consent was obtained from them before inclusion. This research received no grant from any funding agency in the public or commercial sectors.

### 2.1. Statistical Analysis

Statistical analysis was conducted using the SPSS software (version 26.0) (SPSS Inc., Chicago, IL, USA). All participants were chosen by using the systematic random sample technique from the attendees who fulfilled the inclusion criteria. The data were tested for normality using the Kolmogorov–Smirnov test and Shapiro-Wilk test before further statistical analysis; all studied variables were not normally distributed. Differences between groups for continuous measures were analyzed by a Mann–Whitney *U* test and Wilcoxon Signed Ranks Test for categorical measures.

## 3. Results

The study included 45 eyes of 50 subjects who were divided into two groups of patients, the first epiphora group (EG1:25 eyes), who had acquired punctal stenosis and epiphora and managed by simple punctal dilatation. The second epiphora group (EG2:20 eyes) who managed by punctal dilatation augmented with the application of perforated punctal plugs for two months.

Both groups were comparable regarding mean age (39 ± 11 vs 50 ± 12 years, *P* value = 0.4) and sex (males were 36% vs 40%, female were 64% vs 60%, *P* value = 0.5) respectively with no statistically significant difference between both groups (Table 1). Mean duration of epiphora was measured in both groups before treatment (EG1 = 1.656 ± 0.41 months, EG2 = 1.73 ± 0.32 months) with no statistically significant difference (*P* value = 0.436). Various etiologies of acquired Punctal stenosis were summarized in Table 2. The outer diameter and the length of the lower punctum of both groups before treatment and during the followup period comparing both groups were assessed objectively by AS-OCT and summarized in (Tables 3 and 4).

**Figure 1:**
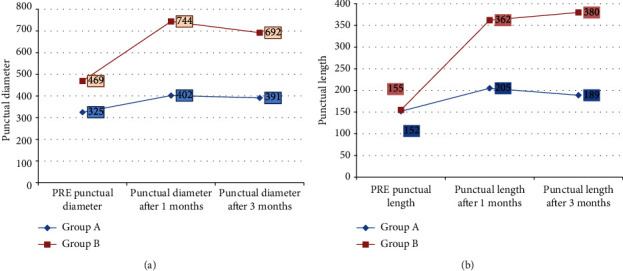
Comparison of Outer Punctal diameter and the length of the lower punctum between both groups before treatment and during the followup period.

**Figure 2:**
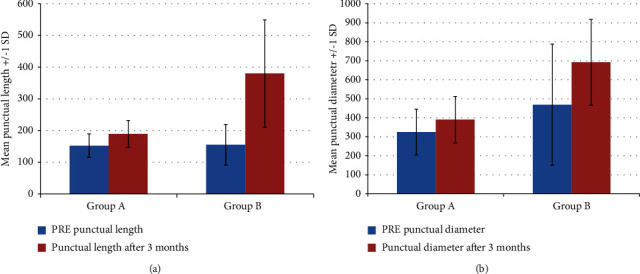
Comparison of the length of the lower punctum and the length of the lower punctum between both groups before treatment and after 3 months.

Both groups were comparable regarding outer punctual diameter and length between the puncti before treatment. Outer punctal diameters (EG1 325 ± 120 um, EG2 469 ± 119 um, *P* value = 0.4). length between outer and inner puncti (EG1 152 ± 37 um, EG2 155 ± 64 um, *P* value = 0.4). There was marked improvement of the outer punctual diameter and length between outer and inner puncti in EG1 (EG1 391 um ± 122 um, EG2 692 ± 226 um (*P* value < 0.007) and EG1 189 ± 43 um, 380 ± 169 um (*P* value < 0.0002) during the followup period. EG2 showed more improvement than EG1 when compared during the followup period in both outer punctual diameter and length between outer and inner puncti with (*P* value < 0.003 and < 0.0002) in favor of EG2. However; both groups show improvement with a statistically significant difference in both groups by using AS-OCT (Figures 1 and 2).

**Figure 3:**
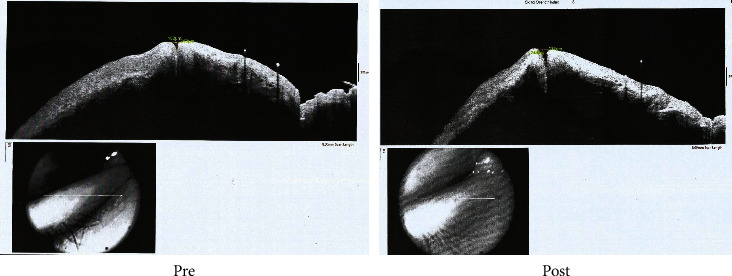
A case of EG1 of simple punctal dilatation before and after treatment.

**Figure 4:**
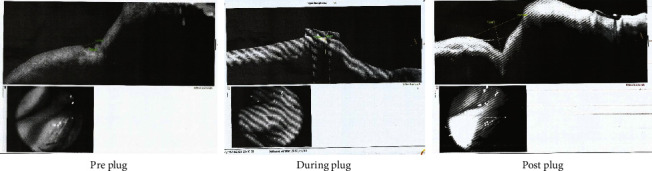
A case of EG2 of punctal dilatation augmented with the application of perforated punctal plugs before, during, and after treatment.

Figures 3 and 4 show AS-OCT of the outer punctual diameter and length between outer and inner puncti in EG1 and EG2.

Subjective assessment of the improvement in both groups using Munk's test was summarized in (Table 5). Both groups showed significant objective improvement using Munk's test, which was more in EG2 when compared to EG1.

## 4. Discussion

In 1989, Bernard et al. [8] described the temporary use of punctal stenting with perforated punctal plugs in cases of acquired punctal stenosis, and several studies after that tried to evaluate the success rates of punctal plug used with promising results. [9, 10] This study is a prospective, interventional, double-blinded controlled randomized study, which was performed on two groups of patients, the first epiphora group (EG1 25 eyes), who had acquired punctal stenosis and epiphora and managed by simple punctal dilatation. The second epiphora group (EG2) who managed by punctal dilatation augmented with the application of perforated punctal plugs for two months. Both groups were evaluated and followed up by using AS-OCT which is a non-invasive imaging method used in previous studies for the evaluation of normal and stenosed punctal [4, 11], and tear meniscus in normal persons. [12] It has been used to examine the proximal lacrimal system, and many studies have investigated the measurement of the anatomical parameters, evaluating the results of punctoplasty, and detecting lacrimal lesions. [13, 14].

Our study aimed to compare simple punctal dilation versus punctal dilation augmented by insertion of PPP with assessing the effect on resolving epiphora and punctum size improvement by an objective and quantitative method, high-resolution AS-OCT imaging to compare punctal parameters of the patients before and after both techniques. The follow-up period was 3 months in both groups of the study with clinical and OCT followup, EG2 showed more improvement than EG1 when compared during the followup period in both outer punctual diameter and length between outer and inner puncti with (*P* value < 0.003 and < 0.0002) in favor of EG2. However; both of the study groups show marked improvement with the statistically significant difference in both groups by using imaging of AS-OCT.

In a study of Abdallah RMA et al, [15] they evaluated the patency and position of perforated lacrimal punctal plugs implanted for treating punctal stenosis together with quantitative assessment of the tear film using AS-OCT, they found that There was a statistically significant postoperative decrease of tear meniscus area and tear meniscus area (*P* < 0.001) and postoperative epiphora Munk score (*P* < 0.001).

Different studies evaluated the efficacy of perforated lacrimal punctal plugs implanted for treating punctal stenosis, in a study of Ozgur et al, [16] they evaluated the efficiency of perforated punctal plugin acquired punctal stenosis, they found epiphora decreased remarkably in 88.9% of the patients 1 month after plug implantation, except one whose plug dropped off spontaneously in 2 weeks. In a study of Chang et al, [17] they reported the success rates for perforated punctual plug (PPP) in the management of acquired punctual stenosis, in a retrospective, cross-sectional, comparative study. They concluded that perforated punctual plug implantation for the treatment of acquired punctual stenosis and obstruction is very effective.

In our study, The plugs were explanted after 2 months with no reported cases of spontaneous extrusion or migration.

Limitations of this study included the small sample size of cases involved in the research (45 eyes), and also, the short period of followup was another limitation (three months), further studies are needed in the future with a larger sample size, and longer followup period to document the efficacy of these plugs in longterm results.

In conclusion; punctal dilation augmented by the insertion of PPP was an effective method in treating cases of inflammatory punctual stenosis as found by monitoring of punctal parameters changes by AS-OCT. AS-OCT was found a useful method for the evaluation of the lacrimal punctal parameters, especially with different treatment modalities in epiphora cases.

## Figures and Tables

**Table 1:** Demographic data and various etiologies of acquired punctal stenosis in both groups.

		EG1, *n* = 25		EG, *n* = 20		*P* Value
Age	Mean + SD	39 ± 11		50 ± 12		0.4
Sex	Male	9	36.0%	8	40.0%	0.5
	Female	16	64.0%	12	60.0%	0.5

**Table 2:** Various etiologies of acquired punctal stenosis in both groups.

Etiology	EG1, *n* = 25	EG2, *n* = 20
Aging	17	15
Inflammatory	5	3
Systemic drug toxicity	1	0
Infectious disease	2	2

**Table 3:** Outer punctual Diameter of both groups during followup three months.

Punctual diameter (In microns)	EG1, *n* = 25					EG2, *n* = 20					*∗P* value
	Mean	±SD	Median	Min	Max	Mean	±SD	Median	Min	Max	
Pre punctual diameter	325	±120	290	149	542	469	±119	402	119	989	0.4
Outer punctual diameter after 1 month	402	±124	393	214	569	744	±224	742	310	1001	<0.001
Outer punctual diameter after 3 months	391	±122	373	200	551	692	±226	724	300	960	<0.007
*∗P*1 value	<0.007					<0.003					

*∗P* value calculates the significant difference of the punctual diameter between the two groups and was calculated by Mann–Whitney *U* test. *∗∗P*1 value calculates the significant difference in punctual Diameter group A and group B before treatment and three months after treatment in each group and was calculated by Wilcoxon Signed Ranks Test.

**Table 4:** Punctual length of both groups during followup three months.

Punctual length(In microns)	EG1, *n* = 25					EG2, *n* = 20					*∗P*-value
	Mean	±SD	Median	Min	Max	Mean	±SD	Median	Min	Max	
Prepunctual length	125	±37	150	102	243	155	±64	134	109	296	0.4
Punctual length after 1 month	205	±45	214	112	265	362	±185	307	165	662	<0.004
Punctual length after 3 months	189	±43	201	109	255	380	±169	308	155	652	<0.0002
*∗∗P*1 value	<0.001					<0.0002					

*∗P*-value calculates the significant difference of the punctual length between the two groups and was calculated by Mann–Whitney *U* test. *P*1 value calculates the significant difference in punctual length in group A and group B before treatment and three months after treatment in each group and was calculated by Wilcoxon Signed Ranks Test.

**Table 5:** Munk's test results of both groups during follow-up three months.

Munk's test	EG1	EG2	*P* value
	*n* = 25	*n* = 20	
	Mean ± SD		
	Median(range)		
Before treatment	3.4 ± 1.1	3.1 + 1	0.09
	4(2–5)	3(1–5)	

One week after treatment	3.1 ± 1	1 ± 1	<0.001
	4(0–4)	2(0–5)	

One month after treatment	2.1 ± 1	1.1 ± 1	<0.001
	2(0–4)	2(1–4)	

Three months after treatment	3.2 ± 1	1.4 ± 1.6	<0.003
	2.5(1–4)	2(0–3)	
*P* Value	<0.002	<0.003	

*∗P*-value calculates the significant difference of the Munk's test between the EG1 and the EG2 and was calculated by Mann–Whitney *U* test, *P*1 value calculates the significant difference in the Munk's test in the EG1 and EG2 group before treatment and 3 months after treatment and was calculated by Wilcoxon Signed Ranks Test.

## Data Availability

The data used to support the results of this study are available from the corresponding author upon request.
